# Tissue Fillers for the Nasolabial Fold Area: A Systematic Review and Meta-Analysis of Randomized Clinical Trials

**DOI:** 10.1007/s00266-021-02439-5

**Published:** 2021-07-13

**Authors:** Tomasz Stefura, Artur Kacprzyk, Jakub Droś, Marta Krzysztofik, Oksana Skomarovska, Marta Fijałkowska, Mateusz Koziej

**Affiliations:** 1 Jagiellonian University Medical College, Świętej Anny 12, 31-008 Kraków Cracow, Poland; 2 Department of Plastic, Reconstructive and Aesthetic Surgery Second Chair of Surgery Medical, University of Lodz, Lodz, Poland; 3 Department of Anatomy, Jagiellonian University Medical College, Cracow, Poland

**Keywords:** Tissue fillers, Dermal fillers, Hyaluronic acid, Nasolabial fold, Meta-analysis

## Abstract

**Abstract:**

Tissue fillers injections remain to be one of the most commonly performed cosmetic procedures. The aim of this meta-analysis was to systematize and present available data on the aesthetic outcomes and safety of treating the nasolabial fold area with tissue fillers. We conducted a systematic review of randomized clinical trials that report outcomes concerning treatment of nasolabial fold area with tissue fillers. We searched the MEDLINE/PubMed, ScienceDirect, EMBASE, BIOSIS, SciELO, Scopus, Cochrane Controlled Register of Trials, CNKI and Web of Science databases. Primary outcomes included aesthetic improvement measured using the Wrinkle Severity Rating Scale score and Global Aesthetic Improvement Scale. Secondary outcomes were incidence rates of complications occurring after the procedure. At baseline, the pooled mean WSRS score was 3.23 (95% CI: 3.20–3.26). One month after the procedure, the pooled WSRS score had reached 1.79 (95% CI: 1.74–1.83). After six months it was 2.02 (95% CI: 1.99–2.05) and after 12 months it was 2.46 (95% CI: 2.4–2.52). One month after the procedure, the pooled GAIS score had reached 2.21 (95% CI: 2.14–2.28). After six months, it was 2.32 (95% CI: 2.26–2.37), and after 12 months, it was 1.27 (95% CI: 1.12–1.42). Overall, the pooled incidence of all complications was 0.58 (95% CI: 0.46–0.7). Most common included lumpiness (43%), tenderness (41%), swelling (34%) and bruising (29%). Tissue fillers used for nasolabial fold area treatment allow achieving a satisfying and sustainable improvement. Most common complications include tenderness, lumpiness, swelling, and bruising.

**Level of Evidence II:**

"This journal requires that authors assign a level of evidence to each article. For a full description of these Evidence-Based Medicine ratings, please refer to the Table of Contents or the online Instructions to Authors  www.springer.com/00266."

**Supplementary Information:**

The online version contains supplementary material available at 10.1007/s00266-021-02439-5.

## Introduction

During aging, the skin undergoes significant changes, collagen becomes fragmented, and its amount decreases; this hinders the interaction between extracellular matrix and fibroblasts, which leads to further deterioration [1]. Various factors can substantially accelerate this process, including ultraviolet exposure leading to rhytids, lentigines, telangiectasias, mottled pigmentation, coarse texture, laxity, and loss of translucency [2]. There are multiple strategies available to prevent and treat premature aging: cosmetological care, topical agents, invasive procedures (i.e., peelings, wrinkle correction, laser rejuvenation), and systemic agents (antioxidants and hormone replacement therapy) [3]. However, the use of tissue fillers remains one of the most performed non-surgical aesthetic procedures in the world [4]. Tissue fillers are currently being used for facial areas (e.g., folds, lip augmentation, depressed scars, enhancement of facial contours), as well as non-facial areas (neck, décolleté, hands) [5]. Most popular tissue fillers include hyaluronic acid (HA), calcium hydroxyapatite (CaHA), collagen-based products (porcine, bovine, and human-derived), and poly-L-lactic acid (PLLA) [6]. Selecting the appropriate filler is crucial in achieving satisfactory, predictable, and sustainable results [7].

The nasolabial fold begins at the junction of the ala nasi, the cheek, and the upper lip and extends in either a straight, convex, or concave shape and ends below and lateral to the corner of the mouth. Its correction was reported to be difficult to achieve by a surgical procedure [8]. Currently, nasolabial fold area wrinkles are most commonly treated with tissue fillers.

Evidence-based medicine (EBM) is currently a gold-standard approach in making decisions concerning the care of individual patients, also in plastic surgery [9]. Despite the importance of EBM, it is only now being introduced into aesthetic medicine [10]. Therefore, it seems timely to conduct a systematic review with meta-analyses to further evaluate available tissue fillers. This will allow us to determine the appropriate treatment for the nasolabial fold area for each patient in the future and assess its safety.

The aim of this systematic review with meta-analysis was to systematize and present available data on the aesthetic outcomes of treating the nasolabial fold area with tissue fillers and compare the effectiveness and safety of various types of tissue fillers.

## Methods

In accordance with the World Medical Association’s Declaration of Helsinki of 2013, the research was registered at PROSPERO. The assigned unique identifying number was “CRD42020219008”. Ethical approval and patient consent were not required for a systematic review using meta-analysis.

### Search Strategy

This study was compliant with the guidelines of the Preferred Reporting Items for Systematic Reviews and Meta-Analyses (PRISMA) (Supplement 1) [11]. Our strategy aimed to find relevant randomized clinical trials investigating the treatment of nasolabial fold area with tissue fillers. A wide search using the MEDLINE/PubMed, ScienceDirect, EMBASE, BIOSIS, SciELO, Scopus, Cochrane Controlled Register of Trials, CNKI, and Web of Science databases was performed until March 3, 2019. The PubMed search strategy is presented in Supplement 2. We did not include any date or language filters. The following terms were combined using Boolean operators “AND” and “OR” and used to conduct a search: “hyaluronic acid”, “dermal filler”, “Hydroxyapatites”, “CaHA”, “Radiesse”, “Polymethyl Methacrylate”, “Injectable filler”, “injectables”, “Polyalkylimide”, “Poly-L-lactic”, “facial”, “nasolabial”, “cosmetic*”, “naso-labial” and “marionette”. We also performed an extensive reference search in the acquired articles for any additional relevant publications.

### Eligibility Assessment

Three independent reviewers performed an eligibility assessment for the relevant full-text articles that were found during the search process. At least two authors assessed each article. We included only prospective randomized clinical trials, reporting data on the treatment of the nasolabial area, which included the Wrinkle Severity Rating Scale (WSRS) score or Global Aesthetic Improvement Scale (GAIS) or complications occurring after the procedure. We excluded studies without a precise description of used dermal filler (for instance, type of the hyaluronic acid used), studies reporting aesthetic improvement results in accordance with other scales than WSRS or GAIS, conference papers, reviews, video articles, case reports, and other studies without relevant data. In case of a lack of agreement between reviewers, a consensus was reached by the whole review team.

### Extraction Strategy

The members of the review team extracted data. Articles in a language other than English were translated into English before the data were extracted. When an assessment of WSRS, GAIS, or complications was conducted by patients and a Medical Doctor/Investigator, results derived from a Medical Doctor/Investigator were considered in the presented meta-analysis. If the assessment of WSRS, GAIS, or complications was conducted by a blinded or a not-blinded investigator, the results from a blinded investigator were included in the meta-analysis.

### Outcomes of Interest

The following data were extracted from these studies: publication year, study design (double-blinded/single-blinded/non-blinded/single-center/multi-center), location (country), follow-up in months, race, sex (% of women), mean/median age, sample (*n*), filler type, filler concentration, the amount of filler being injected, location of injection, needle, eventual touch-up injections, the method of injection, GAIS score, WSRS score, overall complications incidence rate and specific complications incidence rate [redness, bruising, swelling, pruritus, skin induration, skin discoloration, pain, nodulus, hematoma, infection, vascular adverse events (AE), migration, numbness and lumpiness]. We have extracted all complications reported in each study. However, if a specific complication was reported in more than one study, a meta-analysis was conducted.

### Quality Assessment

The Cochrane risk of bias tool (RoB) was used to assess the quality of RCTs included in this meta-analysis. There are seven main domains included in the Cochrane RoB tool: sequence generation, allocation concealment, blinding of participants and personnel, blinding of outcome assessment, incomplete outcome data, selective outcome reports, and other bias. Each domain in each study can be assessed as “high risk of bias”, “low risk of bias”, or “unclear”. Depending on the risk of bias assessment for specific domains overall, each study’s risk of bias was classified as low if all criteria were met (i.e., low risk of bias for each domain) or one criterion was unclear. Alternatively, studies were classified as high risk of bias if one criterion was not met (i.e., high risk of bias for one domain) or two or more criteria were unclear. The risk of bias assessment across studies is presented in Supplementary Material 3.

### Statistical Analysis

Calculations were conducted using MetaXL analysis version 2.0 EpiGear Pty Ltd (Wilston, Queensland, Australia) for the multi-categorical pooled prevalence of different types of complications. Comprehensive Meta-Analysis version 3.0 by Biostat (Englewood, NJ) was used to analyze the morphometric data. The statistical analysis was based on a random-effects model. In case of lacking standard errors or standard deviations of means, they were estimated using a predictive method proposed by Ma et al. [12].

For heterogeneity assessment, the *I*^2 statistics were used, and the results were interpreted as follows: 0–40%, “might not be important”; 30–60%, “could indicate moderate heterogeneity”; 50–90%, “may indicate substantial heterogeneity”; and 75–100% “could represent considerable heterogeneity” [13].

The comparison of confidence intervals for any two pooled means indicated differences between the subgroups, and if they overlapped, the difference was considered statistically insignificant.

## Results

### Acquiring the Studies

**Fig. 1:**
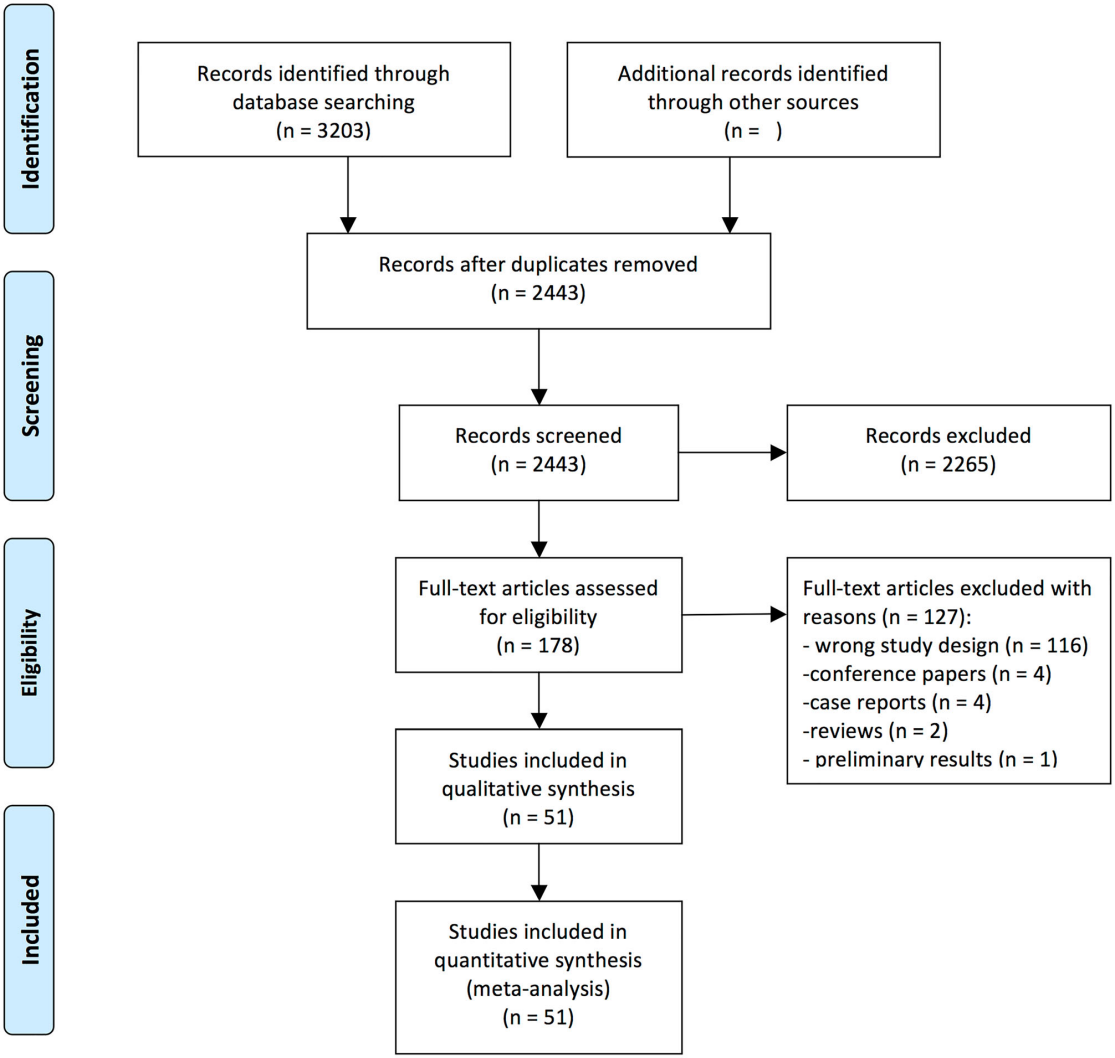
PRISMA flowchart outlining the study inclusion process

Our search strategy resulted in finding 3203 records. Reference screening of those studies did not yield additional articles that met eligibility criteria. After an eligibility assessment, 51 studies were subjected to extraction and quantitative synthesis (meta-analysis). Fig. 1 presents PRISMA Flow-chart outlining the study inclusion process.

### Characteristics of the Included Studies

A total of 4097 patients were included in this meta-analysis. Included studies were published between 2004 and 2019 [14–64]. They were conducted in Canada (two studies, 252 participants), China (four studies, 304 participants), France (two studies, 162 participants), Germany (seven studies, 342 participants), Italy (two studies, 139 participants), Norway (one study, 68 participants), South Korea (13 studies, 749 participants), Spain (one study, 60 participants), Sweden (two studies, 110 participants), Switzerland (one study, 126 participants), the UK (two studies, 80 participants), the United Arab Emirates (one study, 40 participants), and the USA (17 studies, 1945 participants). Overall, 35 studies including 3388 patients were conducted in multiple centers, and 16 single-center studies including 709 patients. WSRS scores were reported by 19 studies, including 1155 patients; GAIS scores were reported by 11 studies including 686 patients; and 39 studies including 3281 patients, reported data on complications. All characteristics of the included studies are presented in Table 1. The quality of the analyzed studies, according to the Cochrane RoB tool, is low.

**Table 1:** Characteristics of the included studies

Author	Publication year	Study design	Location	Multicenter versus single center	Follow-up length	Age*	No patients	Filler	Presented outcomes
Ahn	2012	Double blind	South Korea	multicenter	14 weeks	46.68	40	Mesoglow/IAL System	WSRS/GAIS/Complications
Baumann	2018	Evaluator blind	USA	multicenter	48 weeks	53.7	162	Restylane Defyne/Juvederm Ultra Plus	Complications
Beer	2007	Evaluator blind	USA	single center	6 months	53±12	15	HA/hylan B plus	WSRS/GAIS
Brandt	2010	Double blind	USA	multicenter	2 weeks	53.4±8	60	Perlane—LGP/Perlane –L—LGP with 0.3% lidocaine hydrochloride	Complications
Buntrock	2013	Double blind	Germany	single center	48 weeks	52±5.6	20	CPMHA/biphasic HA filler (NASHA)	WSRS
Carruthers	2005	Double blind	Canada	multicenter	12 months	51,9 (34–83)	150	Restylane Perlane/Hylaform	Complications
Choi	2015	Double blind	South Korea	multicenter	24 weeks	52.48 ± 8.23	66	PP-501-A-Lidocaine/Restylane Lidocaine	WSRS/GAIS/Complications
Dover	2009	Double blind	USA	multicenter	24 weeks	54.05	283	NASHA	Complications
Fagien	2018	Evaluator blind	USA	multicenter	48 weeks	53.9±8.22	170	Juvederm Ultra/Restylane Refyne with lidocaine 3%	Complications
Fino	2019	Double blind	Italy	single center	6 months	50.4±8.3	65	Ial System Duo/Belotero Basic/Balance	Complications
Galadari	2015	Double blind	United Arab Emirates	single center	12 months	46	40	polycaprolactone (PCL)/NASHA	WSRS
Gold	2018	Double blind	USA	multicenter	24 weeks	55.4±10.1	163	Revanesse Versa/Restylane	Complications
Grimes	2009	Double blind	USA	multicenter	25 weeks	50 (32–75)	53	Juvederm Ultra, Juvederm Ultra Plus, Juvederm 30, Hylaform, Hylaform Plus, Captique	Complications
Heden	2010	Double blind	Sweden	multicenter	12 months	54	42	NASHA	Complications
Hong	2018	Patient/evaluator-blinded	South Korea	single center	48 weeks	48.3±7.36	91	DHF-001, Restylane SubQ	WSRS/
Hu	2017	Evaluator blind	China	single center	12 months	(30–65)	57	HA (Nature, Beijing Aimeike Bio-tech Co.), autologous fat injection	WSRS/GAIS/Complications
Hyun	2014	Evaluator blind	South Korea	multicenter	24 weeks	51.9±6.9	58	Restylane, Aesthefill	WSRS/Complications
Joo	2016	Double blind	South Korea	multicenter	24 weeks	47.62±7.64	60	Neuramis, Perlane-L	WSRS/GAIS
Kim	2016	Evaluator blind	South Korea	multicenter	24 months	49.6±11.8	13	HA-G-monophasic, HA-P-biphasic	GAIS/Complications
Lee	2014	Double blind	South Korea	multicenter	12 months	42.2	57	Terafill, Koken	WSRS/GAIS
Levy	2009	Single blind	Switzerland	multicenter		53	126	Juvederm Ultra 3, Restylane Perlane	Complications
Lindqvist	2005	Single blind	Sweden/Norway	multicenter	12 months	49.4	68	Perlane, Zyplast	Complications
Lupo	2007	Double blind	USA	multicenter	24 weeks	49	87	Juvederm Ultra +, Zyplast	WSRS
Marmur	2010	Single blind	USA	single center	1 month		50	CaHA+lidocaine, CaHA	Complications
Moers-Carpi	2007	Evaluator blind	Germany/Spain	multicenter	12 months	50.5 (34–67)	60	CaHA, NASHA	Complications
Monheit	2018	Double blind	USA	multicenter	6 months	54 (33–83)	123	VYC-17.5L (HA+lidocaine)	Complications
Monheit	2010	Patient/evaluator-blinded	USA	multicenter	36 weeks	52.7±9.3	140	DGE, NASHA	Complications
Monheit	2010	Single blind	USA	single center	2 weeks	45 (29–68)	45	Prevelle SILK, Captique	Complications
Moon	2014	Evaluator blind	South Korea	multicenter	6 months	47.2 (21–66)	57	Porcine collagen, Bovine collagen	WSRS/Complications
Narins	2007	Evaluator blind	USA	multicenter	6 months	56	149	Dermicol-P35, NASHA	Complications
Narins	2010	Evaluator blind	USA	multicenter	24 weeks	52.4 (25.7–75.7)	118	CPMHA. bovine collagen	GAIS
Nast	2011	Double blind	Germany	single center	4 weeks	54.8±8.8	60	HA-biphasic, HA-monophasic	WSRS
Onesti	2009	Double blind	Italy	single center	6 months	50.2	74	Captique, Puragen	Complications
Pak	2015	Double blind	South Korea	multicenter	6 months		69	Neuraminis, Restylane	WSRS/Complications
Park	2011	Evaluator blind	South Korea	single center	4 months	48.4±5	12	Teosyal, HA (Titan) + Infrared	WSRS/GAIS/Complications
Park	2015	Double blind	South Korea	multicenter	6 months	45.76±7.77	98	PP-501-B, Restylane Perlane PER	WSRS/GAIS/Complications
Prager	2010	Patient/evaluator-blinded	Germany	single center	1 month	45.8	20	Belotero, Restylane	Complications
Prager	2012	Patient/evaluator-blinded	Germany	single center	12 months	45.8	20	Belotero, Restylane	Complications
Rhee	2014	Evaluator blind	South Korea	multicenter	6 months		68	Elravie, Restylane	WSRS
Rzany	2011	Evaluator blind	France/Germany	multicenter	6 months	50.6±10.1	81	Emervel Classic, Restylane	Complications
Rzany	2017	Patient/evaluator-blinded	France/Germany	multicenter	18 months	50.8	81	Emervel Classic, Restylane	Complications
Schachter	2016	Double blind	Canada	multicenter	1 month	48.84±9.43	102	CaHA (Radiesse) + lidocaine, CaHA (Radiesse)	Complications
Sharma	2011	No blinding	UK	single center	9 months		17	Juvederm Ultra 3, Uma Jeunesse	Complications
Sharma	2018	Single blind	UK	single center	9 months	40.5±11.02	73	Uma Jeunesse Classic, Uma Jeunesse Ultra	Complications
Smith	2007	Evaluator blind	USA	multicenter	6 months	54.7	117	CaHA (Radiesse), human-based collagen (Cosmoplast)	Complications
Suh	2017	Double blind	South Korea	multicenter	24 weeks	46.02±7.21	60	Dermalax implant plus, Restylane Sub-Q	Complications
Taylor	2010	Patient/evaluator-blinded	USA	multicenter	24 weeks		150	Restylane, Perlane	WSRS/GAIS
Weiss	2010	Double blind	USA	multicenter	2 weeks	52.1±6.6	60	Restylane + lidocaine, Restylane	Complications
Wu	2016	Double blind	China	multicenter	15 months	44.7 (24–61)	88	Restylane, BioHyalux	Complications
Wu	2016	Double blind	China	multicenter	6 months	39 (26–58)	109	Restylane, Juvederm Ultra	Complications
Zhou	2016	Double blind	China	single center	24 weeks	47.85	50	Matrifill, Restylane	WSRS

* Age expressed as mean ± standard deviation or median with range or exclusively as mean

### Improvement of the Nasolabial Fold Area—A Meta-Analysis of Reported WSRS Scores

**Fig. 2:**
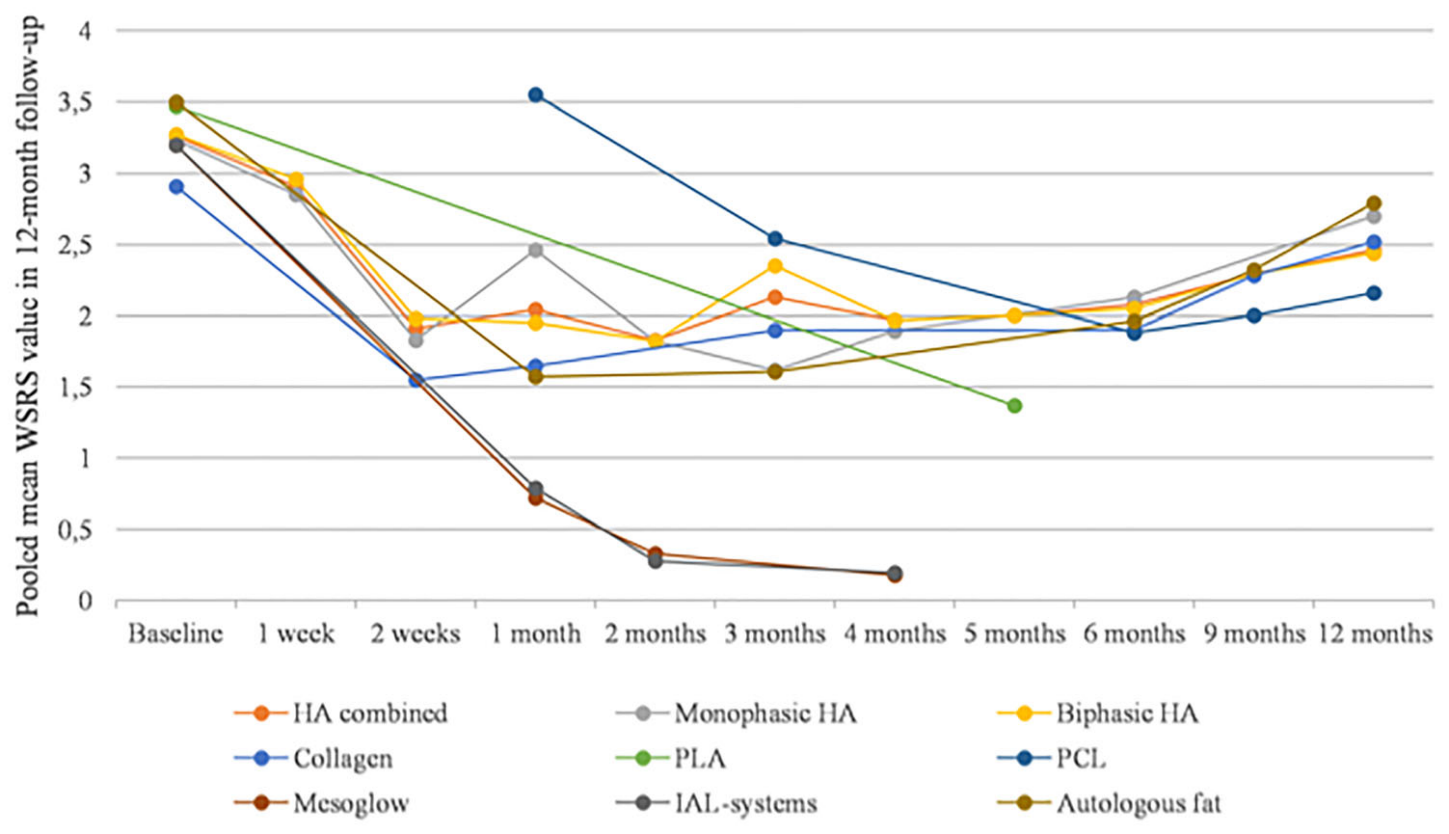
Pooled mean WSRS scores in 12-month follow-up

At baseline, the pooled mean WSRS score was 3.23 (95% CI: 3.20–3.26). After the first week since the injection, it was 2.91 (95% CI: 2.82–2.99). At half-month since the initial procedure, the pooled mean WSRS score had dropped to 1.78 (95% CI: 1.72–1.85). One month after the procedure, the pooled WSRS score had reached 1.79 (95% CI: 1.74–1.83). After 2 months, it was 1.64 (95% CI: 1.6–1.68). At 3-, 4-, and 5-month follow-ups, the reported pooled mean WSRS scores were 2.03 (95% CI: 1.97–2.1), 1.68 (1.65–1.72), and 1.59 (95% CI: 1.42–1.76), respectively. A meta-analysis of longer-term follow-up outcomes revealed pooled mean WSRS scores of 2.02 (95% CI: 1.99–2.05), 2.25 (95% CI: 2.18–2.31), and 2.46 (95% CI: 2.4–2.52) for examinations after 6, 9, and 12 months from the filler injection. Pooled mean WSRS scores for specific groups of tissue fillers are presented in Table 2 and Fig. 2.

**Table 2:** Pooled mean WSRS in 12-month follow-up (pooled means are given as mean with 95% confidence intervals)

	Overall	HA combined	Monophasic HA	Biphasic HA	Collagen	PLA	PCL	Mesoglow	IAL-systems	Autologous fat
Baseline	3.23 [3.20–3.26]	3.26 [3.21–3.3]	3.23 [3.16–3.30]	3.27 [3.22–3.32]	2.91 [2.84–2.99]	3.47 [3.28–3.66]		3.2 [3.07–3.33]	3.2 [3.07–3.33]	3.5 [3.31–3.69]
* I*^2^	93%	95%	94%	95%	0%	NA		NA	NA	NA
No of studies	15	10	6	8	3	1	0	1	1	1
1st week	2.91 [2.82–2.99]	2.91 [2.82–2.99]	2.85 [2.73–2.98]	2.96 [2.84–3.08]						
* I*^2^	99%	99%	100%	100%						
No of studies	2	2	2	2	0	0	0	0	0	0
2 weeks	1.78 [1.72–1.85]	1.91 [1.83–2]	1.83 [1.7–1.95]	1.98 [1.87–2.09]	1.55 [1.43–1.66]					
I^2^	89%	88%	94%	76%	NA					
No of studies	5	4	3	3	1	0	0	0	0	0
1 month	1.79 [1.74–1.83]	2.04 [1.98–2.1]	2.46 [2.31–2.6]	1.95 [1.88–2.02]	1.65 [1.52–1.77]		3.55 [3.4–3.71]	0.72 [0.6–0.85]	0.79 [0.66–0.92]	1.57 [1.39–1.76]
* I*^2^	99%	98%	0%	98%	NA		NA	90%	74%	NA
No of studies	7	4	2	3	1	0	1	1	1	1
2 months	1.64 [1.6–1.68]	1.83 [1.79–1.87]	1.82 [1.77–1.87]	1.82 [1.77–1.87]				0.33 [0.18–0.48]	0.28 [0.12–0.44]	
* I*^2^	98%	88%	91%	84%				NA	NA	
No of studies	8	7	4	6	0	0	0	1	1	0
3 months	2.03 [1.97–2.1]	2.13 [2.03–2.22]	1.62 [1.45–1.79]	2.35 [2.24–2.46]	1.9 [1.79–2]		2.54 [2.33–2.75]			1.61 [1.4–1.82]
* I*^2^	93%	96%	NA	93%	0%		NA			NA
No of studies	6	3	1	3	2	0	1	0	0	1
4 months	1.68 [1.65–1.72]	1.97 [1.93–2.01]	1.89 [1.83–1.95]	1.97 [1.93–2.02]				0.18 [0.06–0.3]	0.2 [0.06–0.34]	
* I*^2^	99%	95%	90%	96%				NA	NA	
No of studies	9	8	4	7	0	0	0	1	1	0
5 months	1.59 [1.42–1.76]	2 [1.72–2.28]		2 [1.72–2.28]		1.37 [1.16–1.58]				
* I*^2^	92%	NA		NA		NA				
No of studies	1	1	0	1	0	1	0	0	0	0
6 months	2.02 [1.99–2.05]	2.08 [2.04–2.11]	2.13 [2.08–2.19]	2.05 [2.01–2.09]	1.9 [1.83–1.98]		1.88 [1.68–2.08]			1.96 [1.67–2.25]
* I*^2^	94%	0%	94%	94%	97%		NA			NA
No of studies	15	12	7	10	3	0	1	0	0	1
9 months	2.25 [2.18–2.31]	2.29 [2.2–2.38]		2.29 [2.2–2.38]	2.28 [2.16–2.39]		2 [1.83–2.17]			2.32 [1.95–2.69]
* I*^2^	40%	0%		0%	0%		NA			NA
No of studies	4	2	0	2	1		1	0	0	1
12 months	2.46 [2.4–2.52]	2.46 [2.38–2.54]	2.7 [2.43–2.97]	2.44 [2.36–2.52]	2.52 [2.4–2.63]		2.16 [1.96–2.36]			2.79 [2.48–3.1]
* I*^2^	84%	88%	NA	90%	0%		NA			NA
No of studies	5	3	1	3	1	0	1	0	0	1

*NA*—not applicable

### Improvement of the Nasolabial Fold Area: A Meta-Analysis of Reported GAIS Scores

**Fig. 3:**
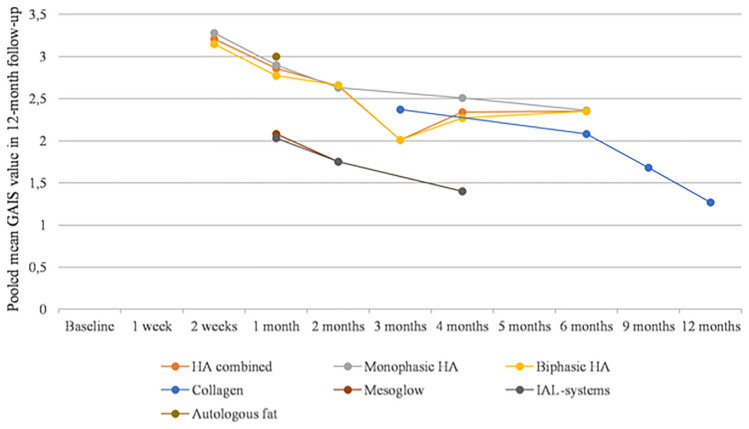
Pooled mean GAIS scores in 12-month follow-up

During follow-up examination after a half-month period since the procedure pooled mean GAIS score was 3.21 (95% CI: 3.11–3.32). After the 1 month since the injection, it was 2.21 (95% CI: 2.14–2.28). Two months after the procedure, the pooled mean GAIS score had reached 2.47 (95% CI: 2.43–2.52). At 3, 4, and 5 months since the treatment, the pooled mean GAIS scores were 2.32 (95% CI: 2.21–2.44), 2.24 (95% CI: 2.2–2.29), and 2.78 (95% CI: 2.6–2.96), respectively. At the 6-, 9- and 12-month time points, the pooled mean GAIS scores were 2.32 (95% CI: 2.26–2.37), 1.68 (95% CI: 1.52–1.83), and 1.27 (95% CI: 1.12–1.42), respectively. Pooled mean GAIS scores for specific groups of tissue fillers are presented in Table 3 and Fig. 3.

**Table 3:** Pooled mean GAIS scores in 12-month follow-up (pooled means are given as mean with 95% confidence intervals)

	Overall	HA combined	Monophasic HA	Biphasic HA	Collagen	Mesoglow	IAL-systems	Autologous fat
2 weeks	3.21 [3.11–3.32]	3.21 [3.11–3.32]	3.28 [3.13–3.43]	3.15 [3.01–3.3]				
* I*^2^	71%	71%	74%	76%				
No of studies	3	3	2	2	0	0	0	0
1 month	2.21 [2.14–2.28]	2.86 [2.55–3.17]	2.9 [2.53–3.28]	2.77 [2.21–3.34]		2.08 [1.97–2.19]	2.03 [1.93–2.14]	3 [2.8–3.2]
* I*^2^	94%	0%	0%	NA		88%	86%	NA
No of studies	4	2	2	1	0	1	1	1
2 months	2.47 [2.43–2.52]	2.65 [2.58–2.71]	2.63 [2.51–2.74]	2.66 [2.58–2.74]		1.75 [1.6–1.9]	1.75 [1.61–1.89]	
* I*^2^	97%	74%	0%	87%		NA	NA	
No of studies	5	4	2	3	0	1	1	0
3 months	2.32 [2.21–2.44]	2.01 [1.68–2.33]		2.01 [1.68–2.33]	2.37 [2.25–2.5]			
I^2^	90%	96%		96%	0%			
No of studies	2	2	0	1	1	0	0	0
4 months	2.24 [2.2–2.29]	2.34 [2.28–2.4]	2.51 [2.39–2.63]	2.27 [2.2–2.35]		1.4 [1.23–1.57]	1.4 [1.25–1.56]	
* I*^2^	98%	98%	50%	98%		NA	NA	
No of studies	6	5	2	4	0	1	1	0
5 months	2.78 [2.6–2.96]							
* I*^2^	NA							
No of studies	1	0	0	0	0	0	0	0
6 months	2.32 [2.26–2.37]	2.35 [2.29–2.42]	2.36 [2.22–2.5]	2.35 [2.28–2.43]	2.08 [1.93–2.24]			
* I*^2^	93%	95%	NA	96%	0%			
No of studies	5	4	1	4	1	0	0	0
9 months	1.68 [1.52–1.83]				1.68 [1.52–1.83]			
* I*^2^	0%				0%			
No of studies	1	0	0	0	1	0	0	0
12 months	1.27 [1.12–1.42]				1.27 [1.12–1.42]			
* I*^2^	0%				0%			
No of studies	1	0	0	0	1	0	0	0

*NA*—not applicable

### Complications After Treatment of the Nasolabial Fold Area

Overall, the pooled incidence of all complications was 0.58 (95% CI: 0.46–0.7). Among studies reporting the overall complications rate, it was 0.59 (95% CI: 0.46–0.72) for all HA fillers, 0.59 (95% CI: 0.36–0.8) for monophasic HA fillers, 0.6 (95% CI: 0.43–0.76) for biphasic HA fillers, 0.4 (95% CI: 0.12–0.7) for all Collagen fillers, 0.82 (95% CI: 0.69–0.93) for Mesoglow, and 0.88 (95% CI: 0.72–0.99) for IAL-systems. Pooled incidences of specific complications (i.e., redness, bruising, swelling, pruritus, skin induration, tenderness, skin discoloration, pain, nodulus, hematoma, infection, vascular adverse events, migration, numbness, and lumpiness) are presented in Table 4.

**Table 4:** Pooled prevalence of complications given as a proportion of patients in which the complication occurred with 95% confidence interval

	Overall	HA combined	Monophasic HA	Biphasic HA	Collagen	PLA	PCL	Mesoglow	IAL-systems	Autologous fat
All complications	0.58 [0.46–0.7]	0.59 [0.46–0.72]	0.59 [0.36–0.8]	0.6 [0.43–0.76]	0.4 [0.12–0.7]			0.82 [0.69–0.93]	0.88 [0.72–0.99]	
*I*^2^	98%	98%	98%	98%	94%			NA	77%	
No of studies	19	17	11	14	2	0	0	1	2	0
Redness	0.26 [0.19–0.33]	0.23 [0.16–0.3]	0.26 [0.14–0.39]	0.2 [0.13–0.29]	0.25 [0.02–0.53]			0.5 [0.34–0.66]	0.43 [0.27–0.58]	0.29 [0.13–0.47]
*I*^2^	97%	96%	97%	96%	97%			NA	NA	NA
No of studies	28	22	13	19	4	0	0	1	1	1
Bruising	0.29 [0.23–0.35]	0.28 [0.22–0.36]	0.29 [0.19–0.4]	0.28 [0.19–0.38]	0.16 [0–0.80]			0.18 [0.07–0.31]	0.27 [0.18–0.36]	0.33 [0.16–0.51]
*I*^2^	95%	95%	93%	95%	99%			NA	0%	NA
No of studies	22	14	11	13	2	0	0	1	2	1
Swelling	0.34 [0.26–0.44]	0.32 [0.23–0.42]	0.41 [0.25–0.57]	0.27 [0.16–0.39]	0.14 [0–0.38]			0.67 [0.52–0.81]	0.65 [0.49–0.79]	0.4 [0.22–0.58]
*I*^2^	98%	98%	97%	98%	97%			NA	NA	NA
No of studies	28	22	12	19	4	0	0	1	1	1
Pruritus	0.17 [0.11–0.22]	0.17 [0.11–0.24]	0.22 [0.13–0.32]	0.13 [0.06–0.22]	0.07 [0–0.2]			0.43 [0.27–0.58]	0.35 [0.21–0.51]	
I^2^	96%	96%	94%	97%	92%			NA	NA	
No of studies	22	18	11	15	3	0	0	1	1	0
Skin induration	0.11 [0.02–0.27]	0.14 [0–0.37]	0.27 [0–0.84]	0.06 [0–0.17]	0.07 [0.02–0.15]					
*I*^2^	97%	98%	98%	93%	78%					
No of studies	7	6	3	4	3	0	0	0	0	0
Tenderness	0.41 [0.21–0.63]	0.39 [0.17–0.64]	0.66 [0.23–1]	0.32 [0.1–0.57]				0.55 [0.39–0.7]	0.53 [0.37–0.68]	*x* [*x*]
I^2^	98%	99%	98%	98%				NA	NA	*x*%
No of studies	7	6	2	5	0	0	0	1	1	*x*
Skin discoloration	0.07 [0.03–0.11]	0.08 [0.03–0.13]	0.14 [0.06–0.24]	0.02 [0–0.05]	0.01 [0–0.04]	0.05 [0–0.15]				
*I*^2^	92%	93%	93%	80%	0%	NA				
No of studies	12	10	7	5	1	1	0	0	0	0
Pain	0.28 [0.2–0.38]	0.31 [0.2–0.41]	0.4 [0.23–0.58]	0.23 [0.12–0.36]	0.07 [0–0.19]			0.57 [0.42–0.73]	0.75 [0.27–1]	0.19 [0.06–0.35]
*I*^2^	98%	98%	98%	98%	89%			NA	95%	NA
No of studies	27	21	13	16	3	0	0	1	2	1
Nodulus	0.05 [0.01–0.1]	0.06 [0.01–0.15]	0.24 [0.0–0.63]	0.02 [0.01–0.03]	0.02 [0.01–0.05]	0.05 [0–0.15]				0.11 [0.02–0.25]
*I*^2^	96%	97%	99%	35%	0%	NA				NA
No of studies	15	9	4	8	2	1	0	0	0	1
Hematoma	0.09 [0–0.23]	0.14 [0–0.23]	0.14 [0–0.39]	0.14 [0–0.52]	0.01 [0–0.02]					
I^2^	97%	96%	95%	97%	NA					
No of studies	6	0	4	4	1	0	0	0	0	0
Infection	0.01 [0–0.03]		0.03 [0–0.10]	0.09 [0.01–0.19]	0.01 [0–0.02]					
*I*^2^	48%		1%	0%	NA					
No of studies	4	0	2	2	1	0	0	0	0	0
Vascular adverse events	0.01 [0–0.02]	0.01 [0–0.02]	0.01 [0.0–0.02]	0.01 [0–0.03]						
I^2^	0%	0%	NA	0%						
No of studies	3	2	1	2	0	0	0	0	0	0
Migration	0.08 [0.01–0.17]		0.08 [0.01–0.17]							
*I*^2^	69%		69%							
No of studies	2	0	2	0	0	0	0	0	0	0
Numbness	0.13 [0.01–0.35]		0.13 [0.01–0.35]							
*I*^2^	92%		92%							
No of studies	3	0	3	0	0	0	0	0	0	0
Lumpiness	0.43 [0.13–0.76]	0.43 [0.13–0.76]	0.34 [0.04–0.72]	0.82 [0.74–0.89]						
*I*^2^	97%	97%	97%	NA						
No of studies	4	4	3	1	0	0	0	0	0	0

*NA*—not applicable

### Injection Technique

Data concerning the injection techniques among included studies are presented in Supplement 4, including injected volume, HA concentration, depth of injection, needle, eventual touch-up injections and the method of injection.

## Discussion

The presented study is one of the first attempts to conduct a comprehensive summary of available randomized clinical trials on tissue fillers, including HA, as well as other fillers, such as collagen, PLA, PCL, Mesoglow, IAL-system, and autologous fat. The focus was on the nasolabial fold, as it is one of the most common locations for tissue fillers injections [65]. Moreover, injecting soft tissue fillers remained one of the most commonly performed cosmetic minimally invasive procedures [66]. Therefore, the relevancy of this meta-analysis cannot be overstated. The search strategy also included marionette folds; however, we did not find available studies to meet our search criteria.

Our results include outcomes on aesthetic improvement measured using WSRS and GAIS scales, as well as a summary of complications following filler injections into the nasolabial fold area. WSRS and GAIS scales were chosen by authors based on the frequent inclusion of them in randomized clinical trials and straightforward interpretation of the results.

We believe that our study is the most comprehensive and current analysis of randomized clinical trials on dermal fillers, conducted according to the EBM principles. To the best of our knowledge, this is the first such comprehensive study to gather and summarize the details of injection techniques and maneuvers used in the nasolabial area.

Although the dermal fillers injections are generally considered safe, some adverse events can occur. Clinicians should have comprehensive knowledge of the possible adverse reactions and be experienced in performing injections with correct technique. Due to the high diversity of available products, the injection techniques may vary. Despite that, the Global Aesthetics Consensus Group attempted to list general principles to minimize the risk of complications [67]. For example, the authors stressed that HA can be administered safely through both needle and cannula; however, it is recommended to use cannulas in the areas susceptible to vascular complications. Care should be taken to aspirate before injection to minimize the risk of intravascular injection. The decision on selection of the appropriate depth of injection should depend on the type of filler and instructions given by manufacturer. In general, HA gels should be injected intradermally or subdermally. It is important not to inject too superficially to avoid the formation of lumps [68]. However, in case of less reticulated gels and/or gels with lower concentrations of HA more superficial injections may be favorable [69]. In the vast majority of included studies clinicians used linear threading or multiple punctures technique and injected HA in the mid- to deep-dermis layer with 27- and 30-gauge needles, what seems to agree with the principles mentioned above.

Among studies included in presented meta-analysis injection volume was in general below 2 ml per nasolabial fold. Most commonly concentration of the HA oscillated around 20 mg/ml. In case of injection technique, in studies included, it was performed most commonly using 27G or 30G needle in the mid-dermal region using methods described as linear threading, single puncture, retrograde injection, etc. Unfortunately, definitions of injection methods are imprecise, often mean the same or differ despite the description used. The statistical analysis of aesthetic outcomes or complications based on injection methods described without a prior precise classification seems to be impossible.

According to the meta-analysis of reported WSRS scores, patients receive immediate significant improvement for any type of filler, which is observable already during the first follow-up appointment. This positive outcome was the most observable for HA, collagen, PLA, PCL, and autologous fat implantation. Improvement in the WSRS score reached its peak approximately 3–5 months after the procedure and then gradually subsided.

According to the results of the GAIS scores meta-analysis, patients also receive a significant improvement after administering HA, collagen, PLA, and autologous fat implantation. Pooled GAIS scores were highest during the first follow-up examination and then progressively decreased.

A previous meta-analysis by Huang et al. concerning safety and efficacy of HA for nasolabial folds reported improvement in the WSRS score at the 6-month follow-up of 1.21 [70]. Our results report a difference of 1.15 between baseline and 6-month follow-up for all HA fillers, which is comparable. The small difference might result from the fact that Huang et al. also included non-randomized studies, which might have heightened the results in their review. A meta-analysis by Wang et al. of randomized clinical trials investigating the treatment of nasolabial folds using HA with lidocaine reported HA with lidocaine is more effective, when dealing with pain after the injection. There was no difference in product effectiveness and safety [71]. We have decided not to analyze the role of lidocaine in our meta-analysis. However, we believe that its impact on aesthetic outcomes and overall safeness is minor, which is confirmed by the study mentioned above.

Overall, the pooled incidence of complications was 58%. The highest reported total complications rates were for Mesoglow (82%) and IAL-system (88%). However, it is important to notice that outcomes of treatment with Mesoglow and IAL-system were reported by very few studies. Analysis of incidence among specific complications revealed that most common are mild, transient, and reversible. They include lumpiness (43%), tenderness (41%), swelling (34%), bruising (29%), pain (28%), and redness (26%). More severe complications that could potentially lead to irreversible damage occur very sporadically. The pooled incidence of infections was 1%, and the pooled incidence of vascular adverse events was also 1%. These results are mostly consistent with previously published research. Abduljabbar et al. reported that injection-related side effects are the most common and usually transient, whereas vascular occlusion is the most severe complication, which is associated with hyaluronic acid filler injection [72]. A meta-analysis by Huang et al. presented incidence rates of specific complications after HA treatment for nasolabial folds, which were comparable to reported in this study: for example, redness (28.7%), swelling (37.3%), bruising (24.7%), and pruritus (11.5%) [70]^.^ Meta-analysis concerning vascular events occurring after facial filler injections by Sito et al. reported that vascular adverse events causing injury to ophthalmic and retinal arteries could result in irreversible damage [73]. To avoid these complications, physicians administering facial tissue fillers injections should have appropriate training and extensive knowledge of facial anatomy with a particular focus, but not limited to vascular anatomy. As Dr. Foad Nahai mentioned in his letter, many patients believe that fillers are 100% safe and tend to overlook potential dangers [74]. There are multiple reports describing cases of vascular occlusion after injecting tissue filler in the nasolabial fold-area [75–77]. The risk seems to be higher in patients with history of cosmetic rhinoplasty [78]. Unfortunately, vascular adverse events associated with potential skin necrosis can occur even, if patients are treated by experienced practitioners. Usually vascular occlusion presents with pain and ischemic pallor but often the symptoms may be atypical [79]. According to Lee et al., Doppler ultrasound could be useful for the prevention of vascular complications during filler injections into nasolabial folds [80]. Introducing immediate treatment in this cases is crucial. Most commonly resolution of symptoms is achieved by administering hyaluronidase [81, 82]. In reality, we do not have successful treatments for complications resulting from vascular occlusion with tissue fillers not treated instantaneously, which can cause blindness, skin necrosis, or stroke.

This systematic review with meta-analysis is associated with several limitations. There is a high level of heterogeneity among the included studies, which certainly limits the precision and generalizability of the results. Studies were conducted in multiple countries, and the methodology used differed substantially. We decided to include as much data as possible to create as many comprehensive reports as possible. Also, we included studies using only the two most common aesthetic improvement scales (WSRS and GAIS). Studies using other scoring systems were excluded, which is a potential source of selection bias. We were also unable to assess the injection method and volume or concentration of the filler used, due to the lack of appropriate information in many available studies. The physician/surgeon’s technical proficiency performing the procedures could also have a great impact on the outcomes of the treatment [83]. Additionally, it is important to consider that several fillers (PLA, PCL, Mesoglow, IAL-systems, and Autologous fat) were underrepresented in published studies. Therefore, results concerning these treatments might be less precise.

Researchers investigating this subject in the future should consider conducting studies using aesthetic improvement measurement methods that are validated and widely used. It is also important to introduce definitions of complications and their classification that would be easy to adhere to and be informative when describing outcomes. In certain available studies, over-correction, under-correction, and lack of satisfaction were classified as complications. In our opinion, the definition of complications in aesthetic medicine procedures should be constructed similarly to those used for surgical research. For instance, complications should be defined as an adverse event that occurred within 6 months from the procedure and is not directly associated with the operative technique. This meta-analysis highlights the need for conducting randomized clinical trials for tissue fillers other than HA, which are under-represented in high EBM level studies.

In conclusion**,** tissue fillers used for nasolabial fold area treatment allow achieving a sustainable (up to 1 year) and satisfying improvement. They are unfortunately associated with complications, most common being tenderness, lumpiness, swelling, and bruising. Most mentioned complications seem to be relatively mild and subside in time.

## Supplementary Information

Below is the link to the electronic supplementary material.

Supplementary file1 (DOC 64 KB)

Supplementary file2 (DOCX 13 KB)

Supplementary file3 (XLSX 16 KB)

Supplementary file4 (DOCX 39 KB)

## Declarations

### Human and animal participants

This article does not contain any studies with human participants or animals performed by any of the authors.
