# Supplementary material for: Tissue Fillers for the Nasolabial Fold Area: A Systematic Review and Meta-Analysis of Randomized Clinical Trials
Source: Aesthetic Plast Surg. 2021 Jul 13;45(5):2300–16. doi: 10.1007/s00266-021-02439-5 (PMC8481177; doi:10.1007/s00266-021-02439-5)
Supplement: Supplementary file 2 — Supplementary file2 (DOCX 13 KB) [file 266_2021_2439_MOESM2_ESM.docx]

**Pubmed Search Strategy:**

(“hyaluronic acid[MeSH Terms]” OR “dermal filler[MeSH Terms]” OR “Hydroxyapatites[MeSH Terms]” OR “CaHA” OR “Radiesse” OR “Polymethyl Methacrylate[MeSH Terms]” OR “Injectable filler*" OR “injectables" OR “Polyalkylimide” OR “Poly-L-lactic”) AND (“facial” OR “face” OR “nasolabial” OR “cosmetic*” OR “naso-labial” OR “marionette*")
