# Supplementary material for: Tissue Fillers for the Nasolabial Fold Area: A Systematic Review and Meta-Analysis of Randomized Clinical Trials
Source: Aesthetic Plast Surg. 2021 Jul 13;45(5):2300–16. doi: 10.1007/s00266-021-02439-5 (PMC8481177; doi:10.1007/s00266-021-02439-5)
Supplement: Supplementary file 4 — Supplementary file4 (DOCX 39 KB) [file 266_2021_2439_MOESM4_ESM.docx]

|  | First author | Filler | Mean injected volume [ml] | HA concentration [mg/ml] | Depth of injection | Needle | Touch-up | Method of injection |
| --- | --- | --- | --- | --- | --- | --- | --- | --- |
| 2012 | Ahn | Mesoglow |  | 20 | mid-dermis | 30G 0.5 inch | after 2 weeks | linear threading |
|  |  | IAL System |  | 20 | mid-dermis | 30G 0.5 inch | after 2 weeks |  |
| 2018 | Baumann | Restylane Defyne | 1.39 | 20 | mid-to-deep dermis |  | after 3 weeks |  |
|  |  | Juvederm Ultra Plus | 1.45 | 24 | mid-to-deep dermis |  | after 3 weeks |  |
| 2007 | Beer | Restylane | 1.4 |  | deep dermis | 30G 0.5 inch |  | linear threading/serial puncture/both |
|  |  | Hylaform | 1.45 |  | dermis | 30G 0.5 inch |  | serial puncture /tunnel |
| 2010 | Brandt | Perlane (LGP-HA) | 1.1 | 20 | deep dermis | 27G 0.5 inch |  | linear threading/ serial puncture/ both |
|  |  | Perlane-L (LGP-HA+L 0,3%) | 1.11 | 20 | deep dermis | 27G 0.5 inch |  | linear threading/ serial puncture/ both |
| 2013 | Buntrock | NASHA | 1 | 20 | deep dermis | 27G | not allowed | serial puncture |
|  |  | CPMHA | 1 | 25 | deep dermis | 27G | not allowed | serial puncture |
| 2005 | Carruthers | NASHA (Perlane) | 1.3 | 20 | mid/deep dermis | 27G, 0.5 inch | 2-week intervals until an optimal cosmetic result was achieved | linear threading/ serial puncture / both |
|  |  | Hylan B (Hylaform) | 1.6 | 5,5 | mid/deep dermis | 30G 0.5 inch | 2-week intervals until an optimal cosmetic result was achieved | linear threading/ serial puncture / both |
| 2015 | Choi | PP-501-A-Lidocaine |  | 33 | mid-dermis | 30G |  | linear threading |
|  |  | RestylaneLidocaine |  | 20 | mid-dermis | 30G |  | linear threading |
| 2009 | Dover | NASHA large gel | 1.23 | 20 | mid-dermis/deep dermis/superficial subcutis | 27G, 0.5 inch | if needed | serial puncture /single puncture with a fanning or threading |
|  |  | NASHA small gel | 1.45 | 20 | mid-dermis/deep dermis/superficial subcutis | 30G 0.5 inch | if needed | serial punctures/single puncture with a fanning or threading |
| 2018 | Fagien | Restylane Refyne with lidocaine 3% | 1.52 | 20 | mid-to-deep dermis |  | 3 weeks after |  |
|  |  | Juvederm Ultra | 1.34 | 24 | mid-to-deep dermis |  | 3 weeks after |  |
| 2019 | Fino | Ial System Duo | 0.89 |  | deep dermis |  | after 30 days |  |
|  |  | Belotero Basic/Balance | 0.84 |  | deep dermis |  | after 30 days |  |
| 2015 | Galadari | Polycaprolactone (PCL) |  |  | mid-dermis | 27 G | after 1 month |  |
|  |  | NASHA |  |  | mid-dermis | 27 G | after 1 month |  |
| 2018 | Gold | Revanesse Versa |  |  | mid-dermis | 27 G | after 1 week | linear threading/fanning |
|  |  | Restylane |  |  | mid-dermis | 27 G | after 1 week |  |
| 2009 | Grimes | Juvederm Ultra | 1.6 | 24 | mid-to-deep dermis |  | 2 at 2 week intervals |  |
|  |  | Juvederm Ultra Plus | 1.6 | 24 | mid-to-deep dermis |  | 2 at 2 week intervals |  |
|  |  | Juvederm 30 | 1.6 | 24 | mid-to-deep dermis |  | 2 at 2 week intervals |  |
|  |  | Hylaform | 2.3 | 5,5 | mid-to-deep dermis |  | not allowed |  |
|  |  | Hylaform Plus | 1.6 | 5,5 | mid-to-deep dermis |  | not allowed |  |
|  |  | Captique | 2 | 5,5 | mid-to-deep dermis |  | not allowed |  |
| 2010 | Heden | NASHA +0.3%lidocaine | 1 | 20 | deep dermis and/or subcutis |  | after 2 weeks | linear threading + fanning |
|  |  | NASHA | 1.1 | 20 | deep dermis and/or subcutis |  | after 2 weeks | linear threading + fanning |
| 2018 | Hong | IDHF-001 | 0,8-1,0 | 20 | the junction of the subcutaneous and deep dermal planes through | 25G |  | linear threading |
|  |  | Restylane Sub-Q | 0,8-1,1 | 20 | the junction of the subcutaneous and deep dermal planes through | 25G |  | linear threading |
| 2017 | Hu | HA (Nature, Beijing, Aimeike Bio-tech Co.) | 0,7-2,2 | 10 | deep dermis and subcutis | 27G 0.5inch | after 1 month | linear threading/serial puncture/both |
| 2014 | Hyun | Aesthefill |  |  | the junction of the subcutaneous and deep dermal planes through | 27G 0.5inch | after 1 month | linear threading |
|  |  | Restylane |  | 20 | subcutaneous and deep dermal planes junction | 27G 0.5inch | after 1 month | linear threading |
| 2016 | Joo | Neuramis |  | 20 | mid-dermis | 27G |  |  |
|  |  | Perlane-L |  | 20 | mid-dermis | 29G |  |  |
| 2016 | Kim | Ha-G-monophasic | 0.64 | 23 | mid-dermis | 27 G | after 2 weeks | linear threading/serial puncture |
|  |  | Ha-P-biphasic | 0.68 | 20 | mid-dermis | 27 G | after 2 weeks | linear threading/serial puncture |
| 2014 | Lee | Therafill | 2.11 |  | mid-dermis/mid-to-upper dermis | 27 G | after 2 weeks | linear threading/fanning |
|  |  | Koken | 2.17 |  | mid-dermis/mid-to-upper dermis |  | after 2 weeks | linear threading/fanning |
| 2009 | Levy | Juvederm Ultra 3(HA+lidocaine) | 0.62 | 24 | mid-to-deep dermis | 27G 0.5inch |  | serial puncture/ fanning |
|  |  | Allergan (HA) | 0.62 | 24 | mid-to-deep dermis | 27G 0.5inch |  |  |
| 2005 | Lindqvist | Perlane | 1.2 | 20 | dermis | 27G 0.5inch | after 2 weeks | linear threading/serial puncture/both |
|  |  | Zyplast | 2.1 | 35 | dermis | 30G 0.5 inch | after 2 weeks | linear threading/serial puncture/both |
| 2007 | Lupo | Juvederm Ultra+ |  | 24 | deep dermis | 27 G | if needed |  |
|  |  | Zyplast |  |  | deep dermis |  | after 2 or 4 weeks |  |
| 2020 | Marmur | CaHA+lidocaine | 0.962 |  |  | 27G |  |  |
|  |  | CaHA | 0.962 |  |  | 27G |  |  |
| 2007 | Moers-Capri | PCL-1 | 1.96 |  | deep dermis | 27G | after 1 month | retrograde injection |
|  |  | PCL-2 | 2.06 |  | deep dermis | 27G | after 1 month | retrograde injection |
| 2016 | Zhou | Matrifill (MMHA) | 0.69 | 16 | mid-to-deep dermis | 30G 0.5 inch | after 4 weeks | retrograde linear threading |
|  |  | Restylane (BHA) | 0.86 | 20 | mid-to-deep dermis | 30G 0.5 inch | after 4 weeks |  |
| 2016 | Wu | Juvederm Ultra | 0.8 | 24 |  |  | 0.3 (0.1-0.5) touch up volume |  |
|  |  | Restylane | 0.8 | 20 |  |  | 0.3 (0.1-0.5) touch up volume |  |
| 2016 | Wu | Restylane | 0.99 | 20 |  |  | after 4 weeks |  |
|  |  | BioHyalux | 0.94 | 20 |  |  | after 4 weeks |  |
| 2010 | Weiss | Restylane + lidocaine | 1.24 | 20 | mid-to-deep dermis | 30G 0.5 inch |  |  |
|  |  | Restylane | 1.23 | 20 | mid-to-deep dermis | 30G 0.5 inch |  |  |
| 2010 | Taylor | Restylane | 2 |  | deep dermis to superficial subcutis |  | after 2 weeks | fanning/ linear threading/serial puncture |
|  |  | Perlane | 2 |  | mid-to-deep dermis |  | after 2 weeks | fanning/ linear threading/serial puncture |
| 2017 | Suh | Dermelax implant plus | 0.8-1.0 | 24 |  | 28G 0.5 inch |  |  |
|  |  | Restylane Sub-Q | 0.8-1.0 | 20 |  |  |  |  |
| 2007 | Smith | Radiesse (CaHA) | 1.2 |  | dermal/subcutaneous junction | 27G 1 1/4-inch | max 2 with 2 weeks interval | linear threading |
|  |  | human-based collagen (Cosmoplast) | 2.4 |  | mid-to-deep dermis | 30 or 31G 0.5 inch | max 2 with 2 weeks interval | linear threading |
| 2018 | Sharma | Uma Jeunesse Classic | 1 |  | mid-to-deepdermis | 27G | no patient required |  |
|  |  | Uma Jeunesse Ultra | 1 |  | mid-to-deep dermis | 27G | no patient required |  |
| 2011 | Sharma | Juvederm Ultra 3 |  |  | deep dermis | 27G |  |  |
|  |  | Uma Jeunesse |  |  | deep dermis | 27G |  |  |
| 2016 | Schachter | Radiesse (CaHA + lidocaine) | 0.84 |  | subdermal plane | 27G 3/4 or 0.5 inch |  | linear threading |
|  |  | Radiesse (CaHA) | 0.83 |  | subdermal plane | 27G 3/4 or 0.5 inch |  | linear threading |
| 2017 | Rzany | Emervel Classic | 0.7 | 20 | mid-dermis | 30G | after 3 weeks, 9 months |  |
|  |  | Restylane | 0.8 | 20 | mid-dermis | 30G | after 3 weeks, 9 months |  |
| 2011 | Rzany | Emervel Classic | 0.9 |  | mid-dermis | 30G |  |  |
|  |  | Restylane | 1 |  | mid-dermis | 30G |  |  |
| 2014 | Rhee | Elravie |  | 23 | mid-dermis | 26 G | after 2 weeks | linear threading |
|  |  | Restylane |  | 20 | mid-dermis | 30 G | after 2 weeks | linear threading |
| 2012 | Prager | Belotero | 0,94-1,88 | 22.5 | mid-to-deep dermis | 27G 0.5-inch |  | linear threading / serial puncture |
|  |  | Restylane | 0,88-1,86 | 20 | mid-to-deep dermis | 27G 0.5-inch |  | linear threading / serial puncture |
|  |  | Juvederm Ultra 3/Juvederm Ultra Plus XC |  | 24 | mid-to-deep dermis | 27G 0.5-inch |  | linear threading / serial puncture |
| 2010 | Prager | Belotero | 1.41 |  | mid-to-deep dermis |  |  | linear threading / serial puncture |
|  |  | Restylane | 1.37 |  | mid-to-deep dermis |  |  | linear threading / serial puncture |
| 2015 | Park | PP-501-B |  | 50 |  | 28 G 0.5 inch |  |  |
|  |  | Restylane Perlane PER |  | 20 |  |  |  |  |
| 2011 | Park | Teosyal |  |  |  | 27G |  |  |
|  |  | Titan (HA) + Infrared |  |  |  | 27G |  |  |
| 2015 | Pak | Neuraminis | <2,0 ml | 20 | dermis | 27G |  |  |
|  |  | Restylane | <2,0 ml | 20 | dermis | 30G |  |  |
| 2009 | Onesti | Captique |  | 5.5 | at the operator’s discretion | 30 G | 2 weeks interval until optimal up to 6 months | linear threading/serial puncture/both |
|  |  | Puragen |  |  | at the operator’s discretion | 30 G | 2 weeks interval until optimal up to 6 months | linear threading/serial puncture/both |
| 2011 | Nast. | HA-biphasic | 1.36 |  | deep dermis |  | after 1 month | tunnel + serial puncture |
|  |  | HA-monophasic | 1.64 |  | deep dermis |  | after 1 month | tunnel + serial puncture |
| 2010 | Narins | CPMHA | 1.16 | 20 |  |  | after 2 weeks | clinical preference of the investigator |
|  |  | bovine collagen | 1.37 | 35 |  |  | after 2 weeks | clinical preference of the investigator |
| 2007 | Narins | Dermicol-P35 | 1.48 | 35 | mid-dermis to lower dermis | 27G 0.5-inch | after 2 weeks | linear threading /serial puncture |
|  |  | NASHA | 1.16 | 20 | mid-dermis to lower dermis | 30G 0.5 inch | after 2 weeks | linear threading /serial puncture |
| 2014 | Moon | porcine collagen | 2.03 |  | mid-dermal level in the upper fold and at the mid- to-upper-dermal level in the lower fold |  | after 2 weeks | fan + linear threading |
|  |  | bovine collagen | 1.87 |  | mid-dermal/mid- to upper-dermal level in the lower fold |  | after 2 weeks | fan + linear threading |
| 2010 | Monheit | Prevelle SILK | 0.95 |  |  |  |  | threading/serial puncture |
|  |  | Captique | 0.8 | 5.5 |  |  |  | threading/serial puncture |
| 2010 | Monheit | DGE | 1.31 | 22 | deep dermis and subcutis | 30G | after 2 and 4 weeks |  |
|  |  | NASHA | 1.52 | 20 | deep dermis and subcutis | 30G | after 2 and 4 weeks |  |
